# Can Oncogenic Animal Viruses Pose a Threat to Humans?

**DOI:** 10.3390/pathogens14111163

**Published:** 2025-11-14

**Authors:** Anna Szczerba-Turek

**Affiliations:** Department of Epizootiology, Faculty of Veterinary Medicine, University of Warmia and Mazury in Olsztyn, Oczapowskiego 13, 10-718 Olsztyn, Poland; a.szczerba@uwm.edu.pl; Tel.: +48-604591361

**Keywords:** oncogenic viruses, zoonotic transmission, viral oncogenesis, cancer, molecular diagnostics, One Health, viral surveillance

## Abstract

Oncogenic viruses are well-established contributors to cancer development in both humans and animals. While many animal oncogenic viruses exhibit strong host specificity, concerns remain about their potential to cross species barriers and impact human health. This article examines the classification and molecular mechanisms of oncogenic viruses, including retroviruses, papillomaviruses, herpesviruses, and hepadnaviruses, in animals. It explores historical cases of cross-species transmission, such as the contamination of early polio vaccines with simian virus 40 (SV40), which resulted from the use of rhesus monkey kidney cells and insufficient screening for latent simian viruses, and the hypothesised association between bovine leukaemia virus (BLV) and human breast cancer. To provide a broader comparative perspective, the discussion also includes examples of viruses with a lower economic impact, illustrating that zoonotic and oncogenic potential is not limited to commercially significant species. Biological barriers—including receptor specificity and immune defences—generally limit transmission; however, frequent human–animal interactions, consumption of contaminated food, and viral mutations may increase zoonotic risk. Advances in molecular diagnostics, such as polymerase chain reaction (PCR), next-generation sequencing (NGS), and serological testing, play a critical role in identifying emerging threats. Prevention strategies, including veterinary vaccination programs, biosafety protocols, and the One Health approach integrating human and veterinary medicine, are essential for mitigating risks. While current evidence indicates that oncogenic animal viruses do not significantly contribute to human cancers, ongoing surveillance and research remain crucial to detect emerging threats. Understanding viral oncogenesis in animals continues to provide valuable insights into cancer prevention and therapy in humans.

## 1. Introduction

Oncogenic viruses, also known as oncoviruses, are biological agents that infect normal cells and disrupt their regulatory mechanisms, resulting in cellular transformation and, potentially, cancer development [1,2,3,4]. Although several viruses are linked to oncogenesis, viral infection alone is rarely sufficient to induce malignant transformation. In most cases, infection constitutes only one of multiple required events, together with host genetic susceptibility, environmental factors, chronic inflammation, or additional somatic mutations that collectively drive tumour initiation. Furthermore, only a small proportion of infected individuals ever develop cancer, and the disease may manifest decades after the initial infection. Understanding this complex and multifactorial relationship between viral infection and cancer is key to avoiding the misconception that viruses directly and uniformly “cause” malignancy [2,4,5].

Oncogenic viruses comprise a diverse group of DNA and RNA viruses that employ distinct molecular strategies to disrupt host–cell regulation. DNA oncogenic viruses—such as *Papillomaviridae* (e.g., human papillomavirus, HPV), *Hepadnaviridae* (e.g., hepatitis B virus, HBV), and *Herpesviridae* (e.g., Epstein–Barr virus, EBV, and Kaposi’s sarcoma-associated herpesvirus, KSHV)—generally induce transformation through the expression of viral oncoproteins that inactivate tumour-suppressor pathways (e.g., p53 and pRb) or by establishing long-term latency accompanied by chronic inflammation [2,3,6,7]. In contrast, RNA oncogenic viruses are primarily members of the *Retroviridae* family (e.g., human T-cell leukaemia virus-1, HTLV-1), which promote cancer by integrating proviral DNA into the host genome, occasionally activating host proto-oncogenes or carrying oncogenes originally captured from host DNA. Other RNA viruses, such as *Flaviviridae* (e.g., hepatitis C virus, HCV), contribute to oncogenesis indirectly via chronic inflammation and oxidative stress rather than integration [8]. Historically, studies on retroviral oncogenes have provided fundamental insight into the molecular basis of cancer [3,4,7].

According to the International Agency for Research on Cancer (IARC), several human viruses have been evaluated and classified as “Group 1 biological agents”—that is, agents that are carcinogenic to humans—based on epidemiological and biological evidence. These include HBV, HCV, twelve mucosal high-risk HPVs (HR-HPVs), EBV (also known as human herpesvirus 4, HHV-4), KSHV (also known as human herpesvirus 8, HHV-8), and HTLV-1 [4,9]. Although human immunodeficiency virus type 1 (HIV-1) plays an indirect role in carcinogenesis, it is also classified by the IARC as a Group 1 agent because virus-induced immunosuppression substantially increases cancer risk in people living with HIV [2,4,9,10].

Oncogenic viruses are responsible for approximately 10–15% of human cancers worldwide [2,9,11]. Comparable oncogenic counterparts exist in animals, such as feline leukaemia virus (FeLV), bovine leukaemia virus (BLV), bovine papillomavirus (BPV), and Marek’s disease virus (MDV), each exploiting specific virus–host interactions to drive tumour formation [12,13,14].

Both in humans and animals, oncogenic viruses induce tumour formation through both direct and indirect mechanisms that disrupt essential cellular regulatory pathways [4]. Direct oncogenesis occurs when viral genomes integrate into host DNA or when viral oncoproteins interfere with critical cell-cycle checkpoints, as observed for FeLV, BLV, BPV [15,16,17,18]. The host is defined as the organism in which viral replication, persistence, or oncogenic transformation occurs. Indirect oncogenesis arises from persistent infection, chronic inflammation, or virus-induced immunosuppression that creates a tumour-promoting microenvironment, as seen with MDV, *Duck hepatitis B virus* (DHBV) [19,20]. Collectively, these mechanisms highlight that viral oncogenesis—whether in animals or humans—is a multistep, multifactorial process shaped by the interplay of viral persistence, immune modulation, and host genetic susceptibility. Recognising the conserved molecular strategies shared across species enhances our understanding of viral evolution, clarifies the zoonotic potential of oncogenic viruses, and informs preventive measures aimed at interrupting the continuum from infection to cancer development.

The zoonotic transmission of viruses is a well-established phenomenon. The emergence of HIV from the Simian Immunodeficiency Virus (SIV) in non-human primates and the spillover of Severe Acute Respiratory Syndrome Coronavirus 2 (SARS-CoV-2) from animal reservoirs exemplify how cross-species transmission can lead to major human disease outbreaks [21,22]. Although most oncogenic viruses exhibit strict host specificity, occasional spillover events—such as the historical contamination of polio vaccines with simian virus 40 (SV40)—illustrate that cross-species exposure is possible [23]. Another debated case is the proposed link between BLV and human breast cancer, for which current evidence remains inconclusive [17,24,25,26,27].

This narrative review critically evaluates the zoonotic potential of animal oncogenic viruses and their possible contribution to human cancers. By analysing viral families, molecular mechanisms, host-switching barriers, and advances in diagnostics and prevention, this article aims to provide a balanced, comprehensive, and scientifically accurate synthesis of current knowledge relevant to One Health. The review addresses existing gaps by integrating cross-species evidence and highlighting novel insights into the mechanisms and emerging risks of viral oncogenesis.

## 2. Oncogenic Viruses in Animals and Animal Models of Oncogenic Virus-Induced Cancers

Oncogenic viruses in animals also represent a diverse group of DNA (*Papillomaviridae*, *Herpesviridae*, *Hepadnaviridae*) and RNA (*Retroviridae*) viruses that utilise distinct molecular and cellular strategies to induce malignant transformation. Comparative studies of these viruses have been instrumental in elucidating universal principles of viral oncogenesis, defining virus–host interactions, and providing experimental models that bridge veterinary and human oncology within the One Health framework. By examining viral mechanisms, host responses, and the translational relevance of animal models, researchers have gained critical insights into the evolution and biology of virus-induced cancers.

Members of the *Papillomaviridae* family, such as BPV and canine papillomavirus (CPV), promote tumorigenesis through the expression of oncoproteins E6 and E7, which inactivate the tumour-suppressor proteins p53 and pRb, respectively [12,28]. These disruptions compromise cell-cycle control and contribute to genomic instability and epithelial transformation. While most papillomavirus infections remain species-restricted and benign, certain BPV types can infect non-bovine hosts such as horses and cats. In these hosts, they cause equine and feline sarcoids, respectively. This is a rare example of natural cross-species transmission [12,14,28,29]. Such cases not only illuminate the role of viral cofactors and host susceptibility in disease progression, but also parallel oncogenic pathways seen in HPV-related malignancies, including cervical and anogenital cancers. Thus, BPV serves as a comparative model to explore E6/E7-mediated oncogenesis and the interplay of host immunity and environmental triggers in papillomavirus-associated cancers [12,18,28,30,31,32,33,34].

*Herpesviridae* have provided a classical model for investigating viral oncogenesis. MDV, a naturally occurring oncogenic herpesvirus in poultry, induces T-cell lymphomas through mechanisms involving viral latency, immune evasion, cytokine dysregulation, and the expression of viral oncogenes such as *Meq*. Studies on MDV have significantly advanced the understanding of latency-associated tumour development, directly informing research on EBV and KSHV in humans [35,36,37,38,39]. Notably, MDV was the first oncogenic virus for which an effective cancer-preventive vaccine was developed, demonstrating that vaccination can successfully prevent virus-induced malignancy. While MDV remains the only example of a vaccine for controlling naturally occurring cancer in animals, similar success has since been achieved in humans with vaccines against oncogenic viruses such as HBV and HPV [40,41]. This model underscores how animal herpesvirus research continues to guide strategies for preventing and controlling human virus-associated cancers [42].

*Hepadnaviridae*, exemplified by DHBV, have provided a powerful model for studying HBV-related hepatocellular carcinoma. Chronic DHBV infection leads to persistent liver inflammation, oxidative stress, and epigenetic reprogramming, reproducing the key steps of HBV-associated carcinogenesis. This system has clarified how viral persistence and host immune responses interact to drive hepatic tumour formation, and it has been critical for evaluating antiviral compounds and vaccine efficacy. Despite species-specific barriers, DHBV models illustrate evolutionary conservation of hepadnaviral replication and its pathogenic outcomes [19,20,43,44].

Among *Retroviridae*, FeLV and BLV serve as classical models for viral oncogenesis. These retroviruses integrate their genomes into host DNA, occasionally activating proto-oncogenes such as *Myc* or disrupting tumour-suppressor loci, thereby driving the development of lymphomas and leukaemias [16,17,45]. Studies on these viruses have been fundamental to elucidating the mechanisms underlying proviral integration, insertional mutagenesis, and retroviral oncogene activation. The discovery that specific retroviruses can capture host oncogenes—first demonstrated in avian and murine systems—proved to be pivotal in the identification of cellular oncogenes and the consequent transformation of our understanding of cancer biology. BLV, in particular, shares mechanistic parallels with HTLV-1, with both deltaretroviruses inducing malignant proliferation through Tax-mediated transcriptional activation and immune dysregulation [46].

Collectively, these animal models underscore the multifactorial nature of viral oncogenesis, where viral persistence, immune modulation, and host genetic context determine disease outcome. Each system offers unique advantages: MDV as a model of latency and vaccination success; BPV as an example of cross-species papillomavirus transmission; DHBV as a model relevant to hepadnaviral liver cancer; and BLV as a parallel to human retroviral leukaemias. Yet, each system also has limitations—such as host specificity and differences in immune response—that must be considered when extrapolating to human disease. By integrating findings across these systems, comparative oncology provides a holistic view of virus-induced tumour biology and highlights conserved molecular pathways that may serve as targets for prevention and therapy in both animals and humans [35].

The comparative overview presented in Table 1 demonstrates that, despite taxonomic and host-specific differences, oncogenic viruses share conserved molecular mechanisms that drive malignant transformation. Across species, common strategies such as the disruption of tumour-suppressor pathways, chronic inflammation, immune modulation, and proviral integration converge to create a tumour-promoting environment. These parallels illustrate the evolutionary conservation of viral oncogenic processes and reinforce the value of animal models in elucidating fundamental cancer mechanisms, evaluating vaccine efficacy, and informing preventive strategies relevant to both veterinary and human medicine within the One Health framework.

Overall, the historical and contemporary data highlight that while several oncogenic viruses have demonstrated cross-species transmission, sustained human adaptation remains rare. This evidence highlights the importance of continuous surveillance of animal reservoirs, as sporadic spillovers may still serve as precursors to oncogenic emergence under changing ecological and biotechnological pressures.

## 3. Potential Risk of Transmission to Humans

The potential for oncogenic animal viruses to cross species barriers and infect humans remains an emerging area of concern within the broader field of zoonotic virology. Four key criteria for assessing such risks can be proposed: (1) experimental evidence of viral entry or replication in human cells or tissues; (2) serological or molecular detection of viral exposure in humans; (3) epidemiological associations between exposure and disease; and (4) ecological or occupational pathways that enable viral transmission. Although most oncogenic viruses exhibit strict host specificity, increased human–animal interactions, together with viral genetic plasticity and immunological vulnerabilities in humans, may occasionally compromise these barriers. Understanding both the biological and ecological determinants of viral spillover is, therefore, essential for anticipating potential threats to public health and for establishing targeted biosurveillance programs.

### 3.1. Biological Barriers and Conditions Facilitating Spillover

The transmission of oncogenic animal viruses to humans is constrained by a series of biological barriers, including host receptor compatibility, intracellular replication competence, and immune system responses. Virus entry is the first major restriction point. For example, members of the family *Retroviridae* such as BLV and FeLV depend on highly specific envelope–receptor interactions that are absent or structurally incompatible in human cells, making productive infection unlikely [16,17,47]. Likewise, *Papillomaviridae* (e.g., BPV) require precise binding to basal epithelial cells for infection, limiting cross-species tropism [14,18,28]. Intracellular replication is a second key barrier; in *Hepadnaviridae*, such as DHBV, replication depends on species-specific polymerases and transcriptional promoters that are non-functional in non-duck cells [19,20].

However, certain biological or environmental factors may weaken these defences and create opportunities for viral adaptation. Immunosuppression—whether due to HIV infection, transplantation, or chemotherapy—can reduce innate antiviral responses, permitting transient replication of otherwise restricted viruses. Co-infections with immunomodulatory viruses such as cytomegalovirus (CMV) or HIV may further suppress interferon-mediated defences, facilitating persistence of foreign viral particles. Experimental data have shown that BLV and FeLV can enter and transiently express proteins in cultured human cells under immunosuppressed conditions, although replication remains incomplete. Genetic mutations and recombination can also alter viral tropism. For example, envelope mutations in retroviruses have been shown to expand receptor usage, while cross-species recombination in papillomaviruses has been associated with altered host range. These findings collectively suggest that while current biological barriers are strong, under specific circumstances of immunological compromise or genetic adaptation, the possibility of oncogenic viral spillover cannot be excluded [48,49].

### 3.2. Historical and Contemporary Evidence of Cross-Species Transmission

Historical cases underscore both the potential and the limitations of zoonotic oncogenic transmission. The most notable example remains SV40, a polyomavirus inadvertently introduced into millions of humans through contaminated polio vaccines between 1955 and 1963 [23]. SV40 can transform human cells in vitro and has been detected in human tumours such as mesothelioma, osteosarcoma, and certain brain cancers. However, large-scale epidemiological studies—including those conducted by the U.S. National Cancer Institute and the World Health Organisation (WHO)—found no consistent increase in cancer incidence among individuals exposed to contaminated vaccines [50,51,52]. Thus, while SV40 exhibits the potential for viral adaptation, its causative role in humans remains unproven.

Another debated example is the proposed link between BLV and human breast cancer. Multiple studies have detected BLV DNA sequences in human breast tissues, with a meta-analysis showing its presence in approximately 59% of malignant tissues compared to 29% of non-malignant samples [17,24]. Similarly, serological evidence of anti-BLV antibodies was reported in dairy farm workers, suggesting occupational exposure [26]. Nonetheless, molecular evidence for a replication-competent virus in humans is lacking, and no direct causative mechanism has been established. Therefore, the current data only support possible exposure but not confirmed infection or oncogenic causality [25,26,53,54,55,56,57].

Other animal oncogenic viruses have exhibited cross-species potential under specific conditions. Simian herpesvirus B (SHBV) can cause fatal encephalitis in humans following exposure to contaminated biological materials or bites from macaques. While SHBV is not classified as an oncogenic virus in humans, its ability to cross species barriers, establish latency, and cause severe pathology illustrates the broader zoonotic risk of herpesviruses. Together, these examples illustrate that although most oncogenic animal viruses remain host-restricted, natural or iatrogenic exposures can sometimes circumvent these boundaries, warranting continued vigilance [35,58,59,60,61]. Additionally, herpesviruses such as MDV and SHBV have demonstrated occasional cross-species transmission. SHBV, for example, has been known to cause fatal encephalitis in humans following exposure to infected macaques. While oncogenic herpesviruses in animals rarely infect humans, their ability to establish latent infections and manipulate host immune responses highlights their potential for zoonotic adaptation [59,60,61].

#### Additional Considerations: Historical and Emerging Biotechnological Contexts

Beyond naturally occurring or accidental exposures, the historical and technological contexts of virology provide further insight into potential zoonotic risks. Similarly to SV40, murine retroviruses—including murine leukaemia virus (MLV)-like and xenotropic murine leukaemia virus-related virus (XMRV) sequences—have occasionally been detected in human tumour or chronic-fatigue-syndrome samples, but subsequent re-examinations attributed most findings to laboratory contamination rather than authentic infection [62,63]. Another debated case involves the detection of mouse mammary tumour virus-like (MMTV-like) sequences in certain human breast cancers. While molecular data suggest possible integration, the absence of a replicating virus and the likelihood of contamination from laboratory reagents remain unresolved issues [25,26,55,64,65].

The increasing use of advanced biotechnologies, such as xenotransplantation, transgenic models, and gene-therapy vectors, introduces new interfaces where viral recombination or cross-species adaptation might occur. Porcine endogenous retroviruses (PERVs), integrated within the pig genome, exemplify this concern. Some PERV-A and PERV-B subtypes can infect human cells in vitro, raising fears of zoonotic transfer during xenotransplantation. However, despite decades of exposure to porcine tissue grafts—including cardiac valves and pancreatic islet cells—no confirmed human infections have been documented. Advances such as CRISPR-Cas9-mediated excision of active PERV loci and the maintenance of donor animals under biosecure, pathogen-free conditions have substantially mitigated this risk [66,67]. Likewise, gene-therapy platforms employing viral vectors derived from animal viruses (e.g., adeno-associated virus from primates, lentivirus from HIV/SIV, or retroviral backbones from murine and avian sources) have revolutionised medicine but continue to raise theoretical concerns regarding insertional mutagenesis, oncogenesis, and unintended host-range expansion. Modern production systems now use replication-incompetent constructs and extensive safety screening to prevent these outcomes, yet continuous monitoring remains essential as vector engineering and cross-species biomedical applications advance [68,69,70] (Table 2).

### 3.3. Factors Increasing the Likelihood of Viral Adaptation and Transmission

The probability of oncogenic animal viruses adapting to humans depends on a complex interplay of exposure intensity, viral mutation rates, and host susceptibility. Occupational exposure represents one of the most significant risk factors: veterinarians, farmers, and slaughterhouse workers are repeatedly exposed to animal blood, secretions, and tissues. In a serological study conducted in Argentina, approximately 37% of dairy workers tested positive for BLV antibodies, compared with 10% of urban controls, suggesting occupational contact as a plausible exposure route [17]. Similarly, butchers and meat-processing workers have shown higher seroprevalence of antibodies against bovine and ovine papillomaviruses, supporting the possibility of cutaneous transmission via microabrasions [18,28]. Dietary practices may also contribute to zoonotic exposure. The consumption of unpasteurised milk and undercooked beef has been associated with the detection of BLV DNA in 30–60% of the tested dairy products, indicating that viral particles can persist through food processing [71]. While infectivity in humans has not been demonstrated, laboratory studies show that papillomaviruses and hepadnaviruses can survive brief exposure to gastric conditions and infect epithelial cells in vitro, suggesting the possibility of oral transmission under favourable conditions [72,73].

Viral evolution further compounds this risk. RNA viruses exhibit high mutation rates that facilitate host-range expansion, while DNA tumour viruses can adapt through recombination and horizontal gene transfer. For example, recombination events in avian leukosis viruses have produced variants capable of infecting new avian species, and similar mechanisms could, in theory, allow papillomaviruses or retroviruses to acquire novel host-adaptation traits [74]. Comparative genomic analyses indicate that several animal oncogenic viruses share 60–85% nucleotide sequence similarity with their human counterparts, reflecting evolutionary continuity that may enable adaptation under selective pressure [13,46].

Overall, these data suggest that the likelihood of zoonotic oncogenic virus transmission is low but not negligible. High-risk occupational groups and immunocompromised populations represent the most vulnerable cohorts. Strengthening viral surveillance in animal reservoirs, monitoring serological evidence of exposure in humans, and adopting One Health frameworks that integrate veterinary, environmental, and medical data are crucial to mitigating the risk of future cross-species viral oncogenesis.

Collectively, the data summarised in Table 3 indicate that the zoonotic transmission of oncogenic animal viruses, although relatively rare, is biologically plausible under conditions of high exposure, immune suppression, or viral adaptation. The convergence of occupational, dietary, and ecological risk factors can create permissive environments for interspecies transmission, particularly when combined with viral mutations that expand the host range. Although definitive evidence of sustained infection or oncogenic transformation in humans remains limited, the recurring detection of viral DNA, antibodies, and sequence homologies highlights the importance of continued vigilance. Integrating molecular surveillance, occupational biosafety, and comparative virology within the One Health framework is essential for the early identification and mitigation of potential viral spillover events [74]. The reviewed mechanisms reveal both shared and virus-specific pathways of oncogenesis, suggesting convergent strategies in host manipulation. Integrating these molecular insights across species enhances understanding of how certain viral traits—such as latency, immune evasion, and integration—may facilitate eventual zoonotic adaptation.

## 4. Advances in Molecular Diagnostics and Surveillance

The continuous evolution of molecular diagnostic technologies has transformed the detection, characterisation, and surveillance of oncogenic viruses. These innovations not only enable early diagnosis in animals and humans but also help identify cross-species transmission events and track viral evolution in real time. Integrating high-sensitivity molecular tools with epidemiological surveillance is essential for assessing the zoonotic potential of oncogenic viruses and implementing timely public health responses.

### 4.1. Molecular Techniques for Detecting Oncogenic Viruses

The detection of oncogenic viruses requires methods capable of identifying low-abundance viral nucleic acids, distinguishing between latent and active infections, and resolving closely related viral strains. The traditional polymerase chain reaction (PCR) and its derivatives—quantitative PCR (qPCR) and reverse transcription PCR (RT-PCR)—remain the foundation of molecular diagnostics. For example, BLV proviral DNA can be detected in peripheral blood with a sensitivity of <10 viral copies per 10^5^ cells, enabling the early identification of infected cattle and even asymptomatic carriers among occupationally exposed humans [17,47,75]. Similarly, HPV genotyping assays targeting the E6/E7 regions can identify up to 30 mucosal HPV subtypes with >98% specificity, facilitating comparative studies of animal and human papillomaviruses [76,77,78,79,80].

Beyond PCR, next-generation sequencing (NGS) and metagenomics have revolutionised oncogenic virus discovery and evolutionary tracing. NGS can generate 10^6^–10^8^ reads per sample, which allows for complete genome reconstruction and the identification of single-nucleotide polymorphisms associated with host adaptation [81]. For example, comparative genomic studies using NGS revealed 85–90% nucleotide similarity between BLV and HTLV-1 tax genes, suggesting evolutionary conservation that may facilitate cross-species adaptation [82,83]. Metagenomic sequencing, applied to blood, milk, and tumour tissues, has uncovered novel papillomavirus and retrovirus strains in livestock that share 60–75% sequence identity with known human oncogenic viruses. However, the use of NGS requires rigorous contamination control and complementary validation via qPCR or serology to distinguish true infections from background noise [84,85].

Additional virus-specific diagnostic tools strengthen detection accuracy. Droplet digital PCR (ddPCR) enables the absolute quantification of integrated viral genomes—such as HPV or HBV DNA—with single-copy precision, while immunohistochemistry (IHC) allows for the spatial localisation of viral proteins (e.g., HPV E6/E7 or MDV Meq) within tumour tissues [86,87,88,89]. Serological assays such as ELISA and Western blot complement nucleic-acid-based methods by identifying virus-specific antibodies. For instance, seroprevalence studies have found BLV antibody rates of 35–40% in dairy workers, compared with less than 10% in controls, providing evidence of occupational exposure [47,90]. Together, these molecular and immunological tools not only enhance diagnostic precision but also enable longitudinal monitoring of viral circulation and adaptation within and between species.

### 4.2. Epidemiological Surveillance of Zoonotic Oncogenic Viruses

Robust surveillance of oncogenic viruses is indispensable for identifying early spillover events and guiding biosafety policies. Current global frameworks led by the WHO, the World Organisation for Animal Health (WOAH), and regional One Health networks support routine molecular screening of high-risk animal reservoirs, including cattle, poultry, and companion animals. Yet, despite these efforts, surveillance remains uneven: fewer than 40% of low-income countries have access to NGS-based viral monitoring, and underreporting in livestock sectors obscures the true prevalence of zoonotic viruses [91,92].

The integration of molecular diagnostics into surveillance systems has begun to yield critical insights. For example, nationwide screening in Japan detected BLV DNA in 34% of dairy herds, correlating with geographic clusters of human BLV seropositivity [93]. In Europe, PCR-based surveys of BPV revealed co-infection rates exceeding 20%, suggesting opportunities for viral recombination that could influence the host range [18]. These findings underscore the need for active molecular surveillance rather than passive case reporting.

To strengthen global readiness, surveillance strategies should prioritise: (1) the establishment of standardised cross-species diagnostic protocols; (2) the expansion of genomic sequencing capacity in veterinary laboratories; (3) the integration of molecular data with clinical and ecological observations; and (4) support for open-access databases on zoonotic virus genomics. Incorporating NGS and metagenomics into field-based programs can enable the early detection of novel oncogenic viruses before they acquire efficient human-to-human transmission potential. Importantly, surveillance must also focus on high-risk populations—farmers, abattoir workers, veterinarians, and immunocompromised individuals—who serve as sentinel groups for potential viral spillover. A globally coordinated One Health surveillance network would, therefore, not only enhance early detection but also reduce long-term economic and public health burdens associated with virus-induced cancers.

The growing precision of molecular diagnostics and the expansion of surveillance networks provide the foundation for effective prevention. The early identification of oncogenic viruses in animal reservoirs enables rapid containment, targeted vaccination, and risk communication before cross-species transmission can occur. Integrating these diagnostic insights into veterinary and public-health policies ensures that preventive measures—such as vaccination, food safety regulation, and biosafety compliance—are evidence-based and timely. Thus, the transition from molecular detection to proactive prevention represents a critical step in minimising the emergence and spread of zoonotic oncogenic viruses. Recent technological advances, particularly in metagenomics and molecular profiling, have expanded the capacity to detect viral sequences with oncogenic potential before clinical disease emerges. However, translating these tools into predictive frameworks for zoonotic oncogenesis requires standardisation, interdisciplinary collaboration, and improved data sharing across human and animal health sectors.

## 5. Prevention Strategies and Public Health Considerations

The effective prevention of zoonotic oncogenic viruses requires a combination of veterinary control, food safety, laboratory biosafety, and sustained public awareness. Preventive efforts must extend beyond animal health to address the economic and social costs of potential cross-species viral oncogenesis.

### 5.1. Preventing Transmission of Oncogenic Animal Viruses

Preventing the spread of oncogenic animal viruses demands a multilayered approach that addresses both biological and behavioural risk factors. Veterinary vaccination programs have demonstrated clear success: immunisation against MDV has reduced poultry lymphoma incidence by over 90% worldwide, and FeLV vaccination has decreased infection rates by 70–80% in domestic cats [94,95,96,97]. These examples underscore the value of expanding vaccine research to livestock oncogenic viruses such as BLV or BPV, which could similarly reduce viral reservoirs [98,99,100].

Food safety measures remain a cornerstone of prevention. Pasteurisation and adequate cooking effectively inactivate BLV and papillomavirus particles, while public education campaigns can limit raw milk and undercooked meat consumption, practices that are still prevalent in many regions [101]. Meanwhile, biosafety in research and diagnostic laboratories is vital: the accidental introduction of SV40 through contaminated polio vaccines highlights how inadequate containment can have global repercussions. Laboratories handling oncogenic viruses should adhere to Biosafety Level 2–3 (BSL-2/3) containment, with rigorous waste management, personnel training, and molecular contamination control.

Ultimately, the prevention of oncogenic viral transmission relies on a coordinated One Health strategy that combines scientific innovation with social responsibility. Strengthening vaccination coverage, improving food safety standards, and ensuring compliance with biosafety regulations not only reduces zoonotic risks but also demonstrates that investment in prevention is far more cost-effective than managing cancer outcomes after spillover occurs.

### 5.2. One Health Approach to Mitigating Viral Risks

The One Health framework underscores the need for interdisciplinary collaboration between veterinary and human health sectors to monitor, assess, and respond to viral threats at the animal–human–environment interface. Establishing integrated surveillance systems enables the early detection and containment of oncogenic viruses with zoonotic potential, thereby reducing the risk of cross-species transmission and subsequent human health impacts [74,102,103]. Livestock and wildlife surveillance using targeted diagnostics and genomic sequencing is essential to detect high-risk strains early, guide interventions, and characterise wildlife reservoirs implicated in zoonotic emergence. Routine diagnostics and genomic sequencing enable the early detection of high-risk strains, guide interventions, and address the critical role of wildlife reservoirs in emerging zoonoses [92,104,105]. Raising public awareness regarding the risks posed by oncogenic viruses is essential to promoting sustainable prevention and control measures. Educational initiatives that emphasise food safety practices, vaccination uptake, and adherence to biosecurity protocols can strengthen community engagement and enhance compliance with surveillance efforts. By fostering a shared sense of responsibility across the public, veterinary, and human health domains, prevention strategies become more effective and resilient.

Through the implementation of comprehensive prevention strategies, the integration of advanced diagnostic technologies, and sustained intersectoral collaboration, public health systems can significantly mitigate the risks posed by oncogenic animal viruses. The One Health paradigm thus offers a robust framework for safeguarding both animal and human health against present and future zoonotic threats. The collective evidence suggests that preventing oncogenic viral spillovers requires integrated One Health strategies that link veterinary, medical, and environmental surveillance. Strengthening cross-sectoral collaboration and prioritising high-risk interfaces could significantly reduce the likelihood of future zoonotic oncogenic events.

## 6. Conclusions

Oncogenic animal viruses represent a unique intersection of virology, oncology, and public health, raising questions about their potential impact on humans. Although biological barriers such as receptor specificity, host restriction, and immune defence currently limit spillover, historical incidents such as SV40 contamination and molecular evidence of BLV in human tissue show that cross-species exposure is possible. The current weight of evidence does not support a major role for animal oncogenic viruses in human cancers, yet the rapid pace of viral evolution, the global nature of food systems, and the intensification of human–animal interactions demand vigilance. Moving forward, three priorities are clear: first, standardising molecular surveillance with contamination controls and open data sharing; second, targeting high-risk occupational and ecological settings for integrated One Health monitoring; and third, leveraging insights from animal oncogenic viruses to advance therapeutic innovations in cancer prevention and immunotherapy. By uniting veterinary and human medicine under a shared research agenda, the study of animal oncogenic viruses can do more than prevent zoonotic threats—it can also enrich our understanding of cancer biology and open new avenues for translational medicine. Sustained interdisciplinary collaboration, global surveillance, and proactive policy will be critical to ensuring early detection, rapid containment, and the informed use of oncogenic virus models in the fight against cancer.

## Figures and Tables

**Table 1:** Comparative overview of oncogenic viruses in humans vs. animals.

Virus Family	Human Oncogenic Viruses (Associated Cancers)	Animal Oncogenic Viruses (Associated Diseases)	Principal Mechanisms of Oncogenesis
*Papillomaviridae*	human papillomavirus (HPV)—cervical, anogenital, and oropharyngeal cancers	bovine papillomavirus (BPV), canine papillomavirus (CPV)—fibropapillomas, sarcoids, squamous cell carcinomas	E6/E7 oncoproteins disrupt cell-cycle control by inactivating tumour suppressors p53 and pRb [12,14,18,28,29,30,31,32,33,34]
*Herpesviridae*	Epstein–Barr virus (EBV), Kaposi’s sarcoma-associated herpesvirus (KSHV)—lymphomas, Kaposi’s sarcoma	Marek’s disease virus (MDV), simian herpesvirus B (SHBV)—lymphomas, encephalitis	Latency, immune modulation, chronic inflammation, expression of viral oncogenes [35,36,37,38,39,42]
*Hepadnaviridae*	hepatitis B virus (HBV)—hepatocellular carcinoma	duck hepatitis B virus (DHBV)—hepatocellular carcinoma in ducks	Chronic inflammation, oxidative stress, and epigenetic alterations (DNA methylation, histone modification) [19,20,43,44]
*Retroviridae*	human T-cell leukaemia virus type 1 (HTLV-1)—adult T-cell leukaemia/lymphoma	feline leukaemia virus (FeLV), bovine leukaemia virus (BLV)—lymphomas, leukaemias	Proviral integration, insertional mutagenesis, proto-oncogene activation (e.g., Myc), Tax-mediated transcriptional activation [16,17,45,46]

**Table 2:** Summary of historical and biotechnology-related examples relevant to oncogenic and zoonotic viral risks.

Virus/Vector Type	Source or Context	Evidence of Human Exposure	Oncogenic and Zoonotic Risks	Current Status/Mitigation
SV40 (simian virus 40)	Contamination of polio vaccines (1950s–1960s) via rhesus monkey kidney cells	Millions vaccinated; viral DNA detected in some human tissue data	Transforms human cells in vitro; no consistent epidemiological link to cancer	No ongoing exposure; modern vaccines produced under stringent biosafety and screening standards [23,50,51,52]
Murine retroviruses (MLV-like, XMRV)	Laboratory contamination, mouse cell lines, xenografts	Sporadic sequence detection in prostate cancer and chronic fatigue syndrome samples	No replication-competent virus confirmed; contamination most likely	Routine molecular controls and reagent screening prevent recurrence [62,63]
MMTV-like sequences	Detected in certain human breast cancers	PCR-based detection in tumour DNA	Controversial; origin may be contamination or cross-species transfer	No replicating virus identified; further studies required [25,26,55,64,65]
Porcine endogenous retroviruses (PERV-A/B)	Xenotransplantation, porcine tissue grafts	In vitro infection of human cells; no in vivo infections	Theoretical risk of recombination or zoonosis	CRISPR-Cas9 excision of active PERVs; pathogen-free pig lines established [66,67]
Gene-therapy viral vectors (retroviral, adenoviral, AAV)	Biomedical biotechnology and clinical gene therapy	Human recipients of viral vector-based therapy	Rare insertional oncogenesis in early trials; no zoonosis	Replication-defective vectors; rigorous preclinical safety testing and biosafety regulation [68,69,70]

**Table 3:** Principal risk factors for the potential zoonotic transmission of oncogenic animal viruses.

Category	Examples/Supporting Evidence	Potential Impact and Mechanistic Explanation
Occupational exposure	Dairy workers, veterinarians, slaughterhouse workers, butchers. BLV antibodies were detected in ~37% of dairy workers vs. 10% of controls [17]	Direct contact with infected blood, milk, or tissues may permit viral entry through cuts or mucosal abrasions. Frequent exposure increases the likelihood of transient infection or immune sensitisation
Dietary exposure	Consumption of unpasteurised milk or undercooked beef. BLV DNA was detected in 30–60% of dairy products; papillomavirus DNA is occasionally found in meat samples [71,72,73]	Ingestion of infectious particles may allow entry through microabrasions in the oral or intestinal mucosa. The resistance of some viral particles to gastric acidity could facilitate limited infection of epithelial cells
Close contact with wildlife	Handling of primates (e.g., *Simian herpesvirus B* from macaques) [35,61]	Direct zoonotic transmission through bites, scratches, or mucosal contact can result in severe disease (e.g., fatal SHBV encephalitis); illustrates potential for herpesvirus spillover
Immunosuppression	HIV infection, post-transplant immunosuppressive therapy [49]	Decreased antiviral immunity and impaired interferon responses may permit transient replication of species-restricted oncogenic viruses, increasing spillover potential
Co-infections with immunomodulatory viruses	Coinfection with CMV, EBV, or HIV [49]	Synergistic immunosuppressive effects can lower host defences, allow the persistence of foreign viral genomes, or promote viral recombination
Viral mutations and recombination	Retroviral envelope mutations; papillomavirus recombination events; horizontal gene transfer in hepadnaviruses [74]	Mutation and recombination can alter receptor specificity, expand the host range, and enhance adaptation to human cells; comparative genomics revealed 60–85% sequence similarity between some animal and human oncogenic viruses
High-intensity animal–human interfaces	Intensive farming, live-animal markets, wildlife trade [17,23,24]	Increased contact frequency raises the probability of viral exposure and mutation–selection cycles conducive to host adaptation
Environmental persistence of viral particles	Viral stability in milk, meat, or laboratory samples [72,73]	Certain non-enveloped viruses (e.g., papillomaviruses) remain infectious for extended periods outside the host, increasing the risk of indirect exposure through contaminated materials

## Data Availability

Not applicable.

## References

[1] Bogolyubova A.V. (2019). Human Oncogenic Viruses: Old Facts and New Hypotheses. Mol. Biol..

[2] zur Hausen H. (2009). The search for infectious causes of human cancers: Where and why. Virology.

[3] de Martel C., Georges D., Bray F., Ferlay J., Clifford G. (2020). Global burden of cancer attributable to infections in 2018: A worldwide incidence analysis. Lancet Glob. Health.

[4] Biological Agents (2012). IARC Monographs on the Evaluation of Carcinogenic Risks to Humans.

[5] zur Hausen H., de Villiers E.M. (2010). Classification of papillomaviruses (PVs) based on 189 PV types and proposal of taxonomic amendments. Virology.

[6] zur Hausen H. (2002). Papillomaviruses and cancer: From basic studies to clinical application. Nat. Rev. Cancer.

[7] Vogt P. (2012). Retroviral oncogenes: A historical primer. Nat. Rev. Cancer.

[8] Stanaway J., Flaxman A., Naghavi M., Fitzmaurice C., Vos T., Abubakar I., Abu-Raddad L., Assadi R., Bhala N., Cowie B. (2016). The global burden of viral hepatitis from 1990 to 2013: Findings from the Global Burden of Disease Study 2013. Lancet.

[9] Grulich A., van Leeuwen M., Falster M., Vajdic C. (2007). Incidence of cancers in people with HIV/AIDS compared with immunosuppressed transplant recipients: A meta-analysis. Lancet.

[10] Bouvard V., Baan R., Straif K., Grosse Y., Secretan B., El Ghissassi F., Benbrahim-Tallaa L., Guha N., Freeman C., Galichet L. (2009). A review of human carcinogens-Part B: Biological agents. Lancet Oncol..

[11] Parkin D. (2006). The global health burden of infection -associated cancers in the year 2002. Int. J. Cancer.

[12] Nasir L., Campo M. (2008). Bovine papillomaviruses: Their role in the aetiology of cutaneous tumours of bovids and equids. Vet. Dermatol..

[13] Lunardi M., Alfieri A.A., Otonel R.A.A., de Alcantara B.K., Rodrigues W.B., de Miranda A.B., Alfieri A.F. (2013). Genetic characterization of a novel bovine papillomavirus member of the Deltapapillomavirus genus. Vet. Microbiol..

[14] Lunardi M., de Alcantara B.K., Otonel R.A.A., Rodrigues W.B., Alfieri A.F., Alfieri A.A. (2013). Bovine Papillomavirus Type 13 DNA in Equine Sarcoids. J. Clin. Microbiol..

[15] Pierangeli A., Antonelli G., Gentile G. (2015). Immunodeficiency-associated viral oncogenesis. Clin. Microbiol. Infect..

[16] Payne L., Nair V. (2012). The long view: 40 years of avian leukosis research. Avian Pathol..

[17] Buehring G., Shen H., Jensen H., Choi K., Sun D., Nuovo G. (2014). Bovine Leukemia Virus DNA in Human Breast Tissue. Emerg. Infect. Dis..

[18] Campo M. (2002). Animal models of papillomavirus pathogenesis. Virus Res..

[19] Funk A., Mhamdi M., Will H., Sirma H. (2007). Avian hepatitis B viruses: Molecular and cellular biology, phylogenesis, and host tropism. World J. Gastrol..

[20] Summers J., Mason W. (1982). Replication of the genome of a hepatitis-B-like virus by reverse transcriptione of an RNA intermediate. Cell.

[21] Sharp P., Hahn B. (2011). Origins of HIV and the AIDS Pandemic. CSH Perspect. Med..

[22] Zhou P., Yang X., Wang X., Hu B., Zhang L., Zhang W., Si H., Zhu Y., Li B., Huang C. (2020). A pneumonia outbreak associated with a new coronavirus of probable bat origin. Nature.

[23] Lane D., Crawford L. (1979). T-antigen is bound to a host protein in SV-40 transformed cells. Nature.

[24] Khatami A., Pormohammad A., Farzi R., Saadati H., Mehrabi M., Kiani S., Ghorbani S. (2020). Bovine Leukemia virus (BLV) and risk of breast cancer: A systematic review and meta-analysis of case-control studies. Infect. Agents Cancer.

[25] Lawson J., Salmons B., Glenn W. (2018). Oncogenic viruses and Breast Cancer: Mouse Mammary Tumor virus (MMTV), Bovine Leukemia virus (BLV), Human Papilloma virus (HPV), and epstein-Barr virus (EBV). Front. Oncol..

[26] Lawson J., Glenn W. (2024). The viral origins of breast cancer. Infect. Agents Cancer.

[27] Moran P., Farias M., Dolcini G., Ceriani M. (2025). Infectivity and persistence of bovine leukemia virus in human breast cells: Assessing a possible zoonotic link to cancer. Vet. Res. Commun..

[28] Campo M.S. (2003). Papillomavirus and disease in humans and animals. Vet. Comp. Oncol..

[29] Munday J.S., Thomson N., Dunowska M., Knight C.G., Laurie R.E., Hills S. (2015). Genomic characterisation of the feline sarcoid-associated papillomavirus and proposed classification as Bos taurus papillomavirus type 14. Vet. Microbiol..

[30] Melo A., Vásquez A., Andana A., Matamala M., Pino T., Guzmán P., Hoffstetter R., Ili C., Brebi P., Roa J. (2014). Genotyping of human papillomavirus in women under 25 years old treated in the screening program for cervical cancer. Rev. Chil. Infect..

[31] Band V., Dalal S., Delmolino L., Androphy E. (1993). Enhanced degradation of p53 protein in HPV-6 and BPV-1 E6-immortalized human mammary epithelial-cells. EMBO J..

[32] Francis D., Schmid S., Howley P. (2000). Repression of the integrated papillomavirus E6/E7 promoter is required for growth suppression of cervical cancer cells. J. Virol..

[33] Araldi R.P., Reis Assaf S.M., de Carvalho R.F., Caldas Rocha de Carvalho M.A., de Souza J.M., Magnelli R.F., Modolo D.G., Roperto F.P., Stocco R.d.C., Becak W. (2017). Papillomaviruses: A systematic review. Genet. Mol. Biol..

[34] Yang A., Peng S., Farmer E., Zeng Q., Cheng M., Pang X., Wu T., Hung C. (2017). Enhancing antitumor immunogenicity of HPV16-E7 DNA vaccine by fusing DNA encoding E7-antigenic peptide to DNA encoding capsid protein L1 of Bovine papillomavirus. Cell Biosci..

[35] Jarosinski K., Tischer B., Trapp S., Osterrieder N. (2006). Marek’s disease virus: Lytic replication, oncogenesis and control. Expert Rev. Vaccines.

[36] Burgess S., Basaran B., Davison T. (2001). Resistance to Marek’s disease herpesvirus-induced lymphoma is multiphasic and dependent on host genotype. Vet. Pathol..

[37] Burgess S., Davison T. (2002). Identification of the neoplastically transformed cells in Marek’s disease herpesvirus-induced lymphomas: Recognition by the monoclonal antibody AV37. J. Virol..

[38] Parcells M., Dienglewicz R., Anderson A., Morgan R. (1999). Recombinant Marek’s disease virus (MDV)-derived lymphoblastoid cell lines: Regulation of a marker gene within the context of the MDV genome. J. Virol..

[39] Parcells M., Burnside J., Morgan R. (2012). Marek’s Disease Virus-Induced T-Cell Lymphomas. Cancer Associated Viruses.

[40] Mironova M., Ghany M. (2024). Hepatitis B Vaccine: Four Decades on. Vaccines.

[41] Malagón T., Franco E., Tejada R., Vaccarella S. (2024). Epidemiology of HPV-associated cancers past, present and future: Towards prevention and elimination. Nat. Rev. Clin. Oncol..

[42] Faiz N., Cortes A., Guy J., Fogle J., Gimeno I. (2016). Efficacy of various Marek’s disease vaccines protocols for prevention of Marek’s disease virus-induced immunosuppression. Vaccine.

[43] Chassot S., Lambert V., Kay A., Trepo C., Cova L. (1993). Duck Hepatitis-B Virus (DHBV) as a model for understanding hepadnavirus neutralization. Archives of Virology Supplementum.

[44] Liu Q., Jia R., Wang M., Huang J., Zhu D., Chen S., Yin Z., Wang Y., Chen X., Cheng A. (2014). Cloning, expression and purification of duck hepatitis B virus (DHBV) core protein and its use in the development of an indirect ELISA for serologic detection of DHBV infection. Arch. Virol..

[45] Frie M., Coussens P. (2015). Bovine leukemia virus: A major silent threat to proper immune responses in cattle. Vet. Immunol. Immunopath..

[46] Aida Y., Murakami H., Takahashi M., Takeshima S. (2013). Mechanisms of pathogenesis induced by bovine leukemia virus as a model for human T-cell leukemia virus. Front. Microbiol..

[47] Buehring G., Shen H., Jensen H., Jin D., Hudes M., Block G. (2015). Exposure to Bovine Leukemia Virus Is Associated with Breast Cancer: A Case-Control Study. PLoS ONE.

[48] Woo S., Fuertes M., Corrales L., Spranger S., Furdyna M., Leung M., Duggan R., Wang Y., Barber G., Fitzgerald K. (2014). STING-Dependent Cytosolic DNA Sensing Mediates Innate Immune Recognition of Immunogenic Tumors. Immunity.

[49] Rouse B., Sehrawat S. (2010). Immunity and immunopathology to viruses: What decides the outcome?. Nat. Rev. Immunol..

[50] Hachana M., Trimeche M., Ziadi S., Amara K., Korbi S. (2009). Evidence for a role of the Simian Virus 40 in human breast carcinomas. BCRT.

[51] Strickler H., Goedert J. (1998). Exposure to SV40-contaminated poliovirus vaccine and the risk of cancer—A review of the epidemiological evidence. Dev. Biol. Satand..

[52] Carbone M., Rizzo P., Pass H. (2000). Simian Virus 40: The link with human malignant mesothelioma is well established. Anticancer Res..

[53] Lauring A., Frydman J., Andino R. (2013). The role of mutational robustness in RNA virus evolution. Nat. Rev. Microbiol..

[54] Amato S., Ramsey J., Ahern T., Rovnak J., Barlow J., Weaver D., Eyasu L., Singh R., Cintolo-Gonzalez J. (2023). Exploring the presence of bovine leukemia virus among breast cancer tumors in a rural state. BCRT.

[55] Blanco R., Quezada-Romegialli C., Muñoz J. (2025). Bovine Leukemia Virus and Human Breast Cancer: A Review of Clinical and Molecular Evidence. Viruses.

[56] Saeedi-Moghaddam F., Mohammaditabar M., Mozhgani S. (2024). Bovine leukemia virus (BLV) and risk of breast cancer; A systematic review and meta-analysis. Retrovirology.

[57] Zhang R., Jiang J., Sun W., Zhang J., Huang K., Gu X., Yang Y., Xu X., Shi Y., Wang C. (2016). Lack of association between bovine leukemia virus and breast cancer in Chinese patients. BCR.

[58] Perelygina L., Patrusheva I., Vasireddi M., Brock N., Hilliard J. (2015). B Virus (Macacine herpesvirus 1) Glycoprotein D Is Functional but Dispensable for Virus Entry into Macaque and Human Skin Cells. J. Virol..

[59] Vasireddi M., Hilliard J. (2012). Herpes B Virus, Macacine Herpesvirus 1, Breaks Simplex Virus Tradition via Major Histocompatibility Complex Class I Expression in Cells from Human and Macaque Hosts. J. Virol..

[60] Katz D., Gowda M., Vasireddi M., Patrusheva I., Seoh H., Filfili C., Wildesl M., Oh J., Hilliard J. (2017). Identification of unique B virus (Macacine Herpesvirus 1) epitopes of zoonotic and macaque isolates using monoclonal antibodies. PLoS ONE.

[61] Kent J., Kang W., Miller C., Fraser N. (2003). Herpes simplex virus latency-associated transcript gene function. J. Neurovirol..

[62] Smith R. (2010). Contamination of clinical specimens with MLV-encoding nucleic acids: Implications for XMRV and other candidate human retroviruses. Retrovirology.

[63] Kearney M., Spindler J., Wiegand A., Shao W., Anderson E., Maldarelli F., Ruscetti F., Mellors J., Hughes S., Le Grice S. (2012). Multiple Sources of Contamination in Samples from Patients Reported to Have XMRV Infection. PLoS ONE.

[64] Zammarchi F., Pistello M., Piersigilli A., Murr R., Di Cristofano C., Naccarato A., Bevilacqua G. (2006). MMTV-Iike sequences in human breast cancer: A fluorescent PCR/laser microdissection approach. J. Pathol..

[65] Lawson J., Glenn W. (2019). Evidence for a causal role by mouse mammary tumour-like virus in human breast cancer. NPJ Brest Cancer.

[66] Denner J. (2021). Porcine Endogenous Retroviruses and Xenotransplantation, 2021. Viruses.

[67] Niu D., Wei H., Lin L., George H., Wang T., Lee H., Zhao H., Wang Y., Kan Y., Shrock E. (2017). Inactivation of porcine endogenous retrovirus in pigs using CRISPR-Cas9. Science.

[68] Geng G., Xu Y., Hu Z., Wang H., Chen X., Yuan W., Shu Y. (2025). Viral and non-viral vectors in gene therapy: Current state and clinical perspectives. Ebiomedicine.

[69] Naldini L. (2015). Gene therapy returns to centre stage. Nature.

[70] Hacein-Bey-Abina S., Garrigue A., Wang G., Soulier J., Lim A., Morillon E., Clappier E., Caccavelli L., Delabesse E., Beldjord K. (2008). Insertional oncogenesis in 4 patients after retrovirus-mediated gene therapy of SCID-X1. J. Clin. Investig..

[71] Rodríguez S., Florins A., Gillet N., de Brogniez A., Sánchez-Alcaraz M., Boxus M., Boulanger F., Gutiérrez G., Trono K., Alvarez I. (2011). Preventive and Therapeutic Strategies for Bovine Leukemia Virus: Lessons for HTLV. Viruses.

[72] Gonzalez N., Marques M., Domingo J.L. (2021). Respiratory viruses in foods and their potential transmission through the diet: A review of the literature. Environ. Res..

[73] Pomeroy L., Bjornstad O., Holmes E. (2008). The evolutionary and epidemiological dynamics of the paramyxoviridae. J. Mol. Evol..

[74] Destoumieux-Garzón D., Mavingui P., Boetsch G., Boissier J., Darriet F., Duboz P., Fritsch C., Giraudoux P., Le Roux F., Morand S. (2018). The One Health Concept: 10 Years Old and a Long Road Ahead. Front. Vet. Sci..

[75] Higuchi R., Dollinger G., Walsh P., Griffith R. (1992). Simultaneous amplification and detection of specific DNA sequences. Biotechnology.

[76] Mullis K., Faloona F. (1987). Specific syntesis of DNA invitro via a polymerase-catalyzed chain reaction. Method. Enzymol..

[77] Brandwein M., Nuovo G., Biller H. (1993). Analysis of prevalence of human papillomavirus in laryngeal carcinomas—Study of 40 cases using polymerase chain reaction and consensus primers. Ann. Otol. Rinol. Laryngol..

[78] Hesselink A., Berkhof J., van der Salm M., van Splunter A., Geelen T., van Kemenade F., Bleeker M., Heideman D. (2014). Clinical Validation of the HPV-Risk Assay, a Novel Real-Time PCR Assay for Detection of High-Risk Human Papillomavirus DNA by Targeting the E7 Region. J. Clin. Microbiol..

[79] Jacquin E., Saunier M., Mauny F., Schwarz E., Mougin C., Prétet J. (2013). Real-time duplex PCR for simultaneous HPV 16 and HPV 18 DNA quantitation. J. Virol. Methods.

[80] Dagalp S.B., Dogan F., Farzani T.A., Salar S., Bastan A. (2017). The genetic diversity of bovine papillomaviruses (BPV) from different papillomatosis cases in dairy cows in Turkey. Arch. Virol..

[81] Mardis E. (2008). Next-generation DNA sequencing methods. Annu. Rev. Genomics Hum. Genet..

[82] Kato J., Masaki A., Fujii K., Takino H., Murase T., Yonekura K., Utsunomiya A., Ishida T., Iida S., Inagaki H. (2016). Quantitative PCR for HTLV-1 provirus in adult T-cell leukemia/lymphoma using paraffin tumor sections. Pathol. Int..

[83] Ji H., Chang L., Yan Y., Wang L. (2023). Development and validation of a duplex real-time PCR for the rapid detection and quantitation of HTLV-1. Virol. J..

[84] Geoghegan J., Duchêne S., Holmes E. (2017). Comparative analysis estimates the relative frequencies of co-divergence and cross-species transmission within viral families. PLoS Pathog..

[85] Flippot R., Malouf G., Su X., Khayat D., Spano J. (2016). Oncogenic viruses: Lessons learned using next-generation sequencing technologies. Eur. J. Cancer.

[86] Hama H., Aboudharam G., Barbieri R., Lepidi H., Drancourt M. (2022). Immunohistochemical diagnosis of human infectious diseases: A review. Diagn. Pathol..

[87] Qvick A., Andersson E., Almeren A., Waenerlund M., Stenmark B., Karlsson C., Karlsson M., Helenius G. (2024). Sensitive and Specific Droplet Digital PCR Assays for Circulating Tumor HPV DNA: Development, Validation, and Clinical Application in HPV-Associated Cancers. Mol. Diagn. Ther..

[88] Bhambhani C., Sandford E., Haring C., Brummel C., Tuck K., Olesnavich M., Bhangale A., Walline H., Dermody S., Spector M. (2023). Development of a high-performance multi-probe droplet digital PCR assay for high-sensitivity detection of human papillomavirus circulating tumor DNA from plasma. Oral Oncol..

[89] Larsson G., Helenius G. (2017). Digital droplet PCR (ddPCR) for the detection and quantification of HPV 16, 18, 33 and 45-a short report. Cell. Oncol..

[90] Engvall E. (2010). Citation Classic The ELISA, Enzyme-Linked Immunosorbent Assay. Clin. Chem..

[91] Payne L. (1998). Retrovirus-induced disease in poultry. Poult. Sci..

[92] Karesh W., Dobson A., Lloyd-Smith J., Lubroth J., Dixon M., Bennett M., Aldrich S., Harrington T., Formenty P., Loh E. (2012). Zoonoses 1 Ecology of zoonoses: Natural and unnatural histories. Lancet.

[93] Sahashi Y., Oshima M., Yamagishi J., Muramatsu C., Shimizu K., Inoshima Y. (2021). Bovine leukemia virus genotype surveillance in cattle at a slaughterhouse in Aichi Prefecture, Japan, in 2019 using polymerase chain reaction combined with restriction fragment length polymorphism. J. Vet. Med. Sci..

[94] Stern A., Markel H. (2005). The history of vaccines and immunization: Familiar patterns, new challenges. Health Aff..

[95] Hirai K., Sakaguchi M. (2001). Polyvalent recombinant Marek’s disease virus vaccine against poultry diseases. Marek’s Dis. Curr. Top. Microbiol. Immunol..

[96] Witter R. (2001). Protective efficacy of Marek’s disease Vaccines. Marek’s Dis. Curr. Top. Microbiol. Immunol..

[97] Marciani D., Kensil C., Beltz G., Hung C., Cronier J., Aubert A. (1991). Genetically-engineered subunit vaccine against feline leukaemia virus—Protective immune response in cats. Vaccine.

[98] Jin X., Cowsert L., Marshall D., Reed D., Pilacinski W., Lim L., Jenson A. (1990). Bovine serological response to a recombinant BPV-1 major capsid protein vaccine. Intervirology.

[99] Chu P., Chen S., Zhou X., Wei Z., Zhai S. (2025). Getah Virus: A New Contaminant in Veterinary Vaccines. Vet. Sci..

[100] Abdala A., Alvarez I., Brossel H., Calvinho L., Carignano H., Franco L., Gazon H., Gillissen C., Hamaidia M., Hoyos C. (2019). BLV: Lessons on vaccine development. Retrovirology.

[101] Scallan E., Hoekstra R., Angulo F., Tauxe R., Widdowson M., Roy S., Jones J., Griffin P. (2011). Foodborne Illness Acquired in the United States-Major Pathogens. Emerg. Infect. Dis..

[102] Adisasmito W.B., Almuhairi S., Behravesh C.B., Bilivogui P., Bukachi S.A., Casas N., Becerra N.C., Charron D.F., Chaudhary A., Zanella J.R.C. (2022). One Health: A new definition for a sustainable and healthy future. PLoS Pathog..

[103] Rabinowitz P., Conti L. (2013). One Health and Emerging Infectious Diseases: Clinical Perspectives. One Health: The Human-Animal-Environment Interfaces in Emerging Infectious Disease: The Concept and Examples of a One Health Approach.

[104] Judson S., Rabinowitz P. (2021). Zoonoses and global epidemics. Curr. Opin. Infect. Dis..

[105] Karesh W., Cook R. (2005). The human-animal link. Foreign Aff..

